# Spontaneous rupture of a mycotic splenic artery pseudoaneurysm secondary to histoplasmosis: a case report

**DOI:** 10.1186/s40792-024-01920-y

**Published:** 2024-06-03

**Authors:** Mitchell H. Mirande, Dante L. S. Souza, Louis Thibodeaux, Cody Sutphin

**Affiliations:** 1 Department of Surgery, TriHealth, Good Samaritan Hospital, Cincinnati, OH USA; 2 Department of Surgery, TriHealth, Bethesda North Hospital, Cincinnati, OH USA

**Keywords:** Splenic artery aneurysm, Mycotic aneurysm, Pseudoaneurysm, Histoplasmosis, Open splenectomy, Ruptured pseudoaneurysm

## Abstract

**Background:**

A splenic artery pseudoaneurysm is a rare pathology that occurs mainly secondary to pancreatitis, abdominal trauma, peptic ulcers, pancreatic and gastric cancers, and infections. It is best diagnosed using computed tomography angiography and typically treated using endovascular embolization and, in some cases, open or laparoscopic surgery. In this report, we present a case of a ruptured mycotic splenic artery pseudoaneurysm containing Histoplasma capsulatum, which to our knowledge is the first case to report a mycotic splenic artery pseudoaneurysm of this nature.

**Case presentation:**

We report a case of a 42-year-old white male with past medical history of Hepatitis C and IV drug abuse who presented to the Emergency Department with a 24-h history of severe diffuse abdominal pain. He was tachycardic and peritonitic on exam. Work-up demonstrated leukocytosis and lactic acidosis. Computed tomography of the abdomen and pelvis with intravenous contrast showed hemoperitoneum and active extravasation of contrast from the splenic artery into the splenic hilum, associated with a surrounding hematoma measuring 5.3 × 5.0 cm, concerning for ruptured splenic artery pseudoaneurysm. The patient was taken emergently for exploratory laparotomy, where a large intraperitoneal hematoma was evacuated. A ruptured splenic artery pseudoaneurysm was identified, isolated, and controlled, followed by completion splenectomy. Final pathology demonstrated a 3.0 × 1.3 × 0.3 cm pseudoaneurysm wall and a 14 × 9.5 × 5.5 cm spleen containing multiple necrotizing granulomata positive for the presence of Histoplasmosis species. The patient recovered appropriately and was discharged on post-operative day five.

**Conclusions:**

This case demonstrates a successful approach to a ruptured mycotic splenic artery pseudoaneurysm resulting in a positive outcome. It is a unique case as it highlights, to our knowledge, the first report of splenic artery aneurysm secondary to Histoplasma capsulatum infection. This report helps further the understanding of the pathophysiology as well as the natural history of mycotic splenic pseudoaneurysms.

## Background

Splenic artery pseudoaneurysms (SAPs) are rare and occur mainly secondary to pancreatitis, abdominal trauma, gastric ulcers, pancreatic and gastric cancers, and infections (especially in IV drug users) [1–8].They are best diagnosed using computed tomography angiography (CTA) [3, 4, 9–11] and best treated using endovascular embolization [4, 9, 10] but, in some cases open or laparoscopic surgical intervention is necessary [4, 9, 12]. Ruptured SAPs are an important differential diagnosis to consider when a patient presents with abdominal pain, hemoperitoneum, hematemesis, or hematochezia in the setting of the previously mentioned risk factors due to its high risk of mortality (> 90%) if left untreated [3–6, 8, 9, 13, 14].

In this report, we present a case of a ruptured mycotic SAP secondary to Histoplasma species, which to our knowledge is the first of this nature to be reported in the literature. Additionally, we review the presentation, work-up, and current management recommendations when faced with a splenic artery pseudoaneurysm.

## Case presentation

**Fig. 1:**
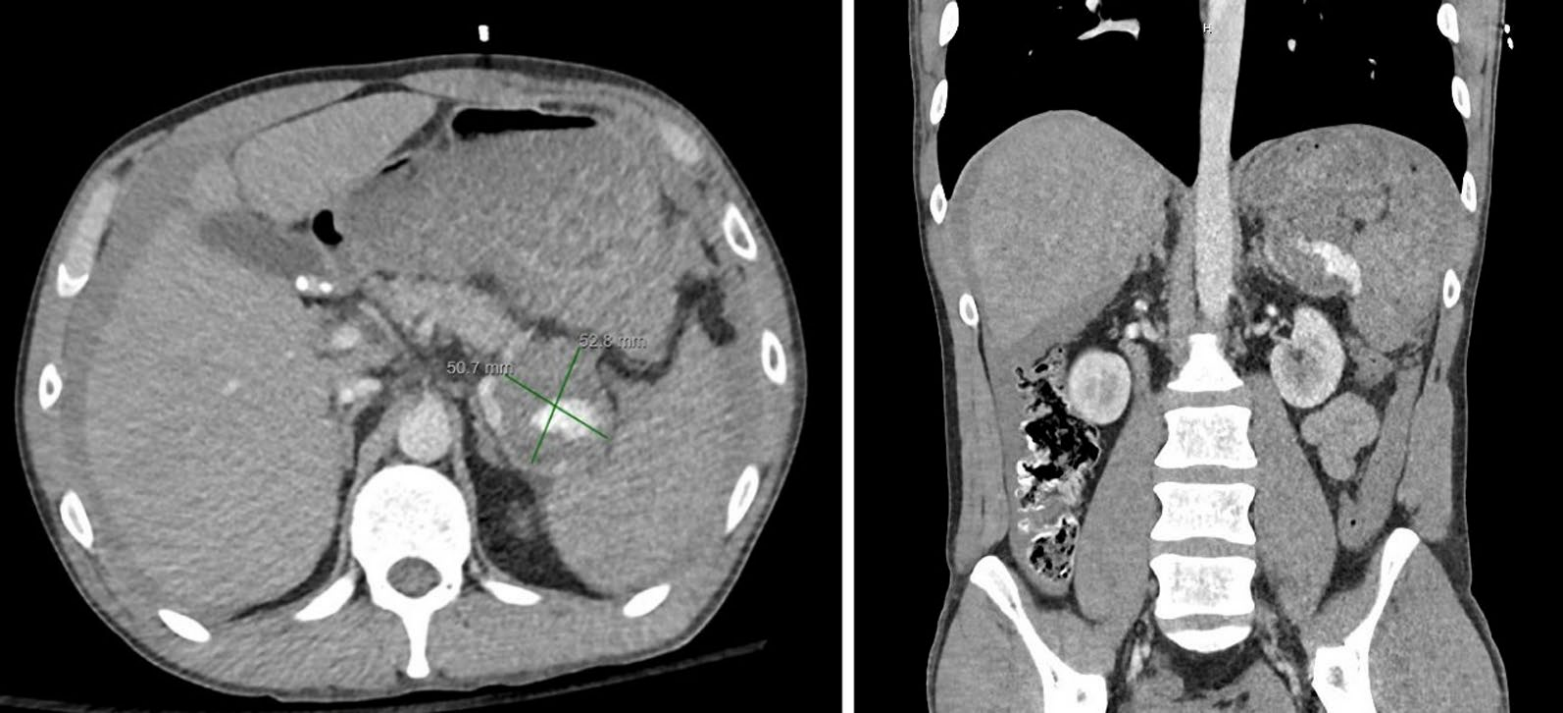
Ruptured splenic artery pseudoaneurysm with active extravasation. (Left) axial view; (right) coronal view

We report a case of a 42-year-old white male with past medical history of Hepatitis C and IV drug abuse who presented with the Emergency Department (ED) with a 24-h history of severe diffuse abdominal, associated with nausea, weakness, lightheadedness, and one episode of syncope. He denied prior history of cancer, pancreatitis or abdominal trauma. On presentation, vital signs were notable for Heart Rate (HR) 140 bpm, Blood Pressure (BP) 103/76 mmHg, Respiratory Rate (RR) 24 bpm, Temperature 36.6C, and SatO2 100% on room air. BMI 22.77 kg/m2. On physical exam, the patient appeared in acute distress, with a rigid and diffusely tender abdomen associated with rebound tenderness and voluntary guarding, consistent with peritonitis. Initial work-up demonstrated Hgb 13.5 g/dL, WBC 15.2 THOU/mcL, Platelets 212 THOU/mcL, Na 137 mEq/L, K 4.0 mEq/L, Creatinine 1.34 mg/dl, BUN 15 mg/dL, Glucose 244 mg/dL, Lactate 2.9 mmol/L, Lipase 53 U/L, Total Bilirubin 1.2 mg/dL, Direct Bilirubin 1.2 mg/dL, ALT 60 IU/L, and AST 50 IU/L. Blood cultures were obtained. Imaging included Computed Tomography (CT) of the Abdomen and Pelvis with intravenous (IV) contrast that demonstrated hemoperitoneum and active extravasation of contrast from the splenic artery into the splenic hilum, associated with a surrounding hematoma measuring 5.3 × 5.0 cm immediately subjacent to the pancreatic tail, concerning for ruptured SAP (Fig. 1). In addition, the spleen was noticed to have a patchy, irregular enhancement (Fig. 1). In the ED, two liters of lactated ringers were administered, and patient remained tachycardic with HR 130 bpm and BP 99/69 mmHg. Interventional Radiology was contacted for consideration of emergent embolization of the splenic artery. However, IR deemed that embolization was relatively contraindicated given patient’s clinical condition (persistent tachycardia and hypotension despite fluid resuscitation) and the location of active extravasation involving the splenic hilum extending to the main splenic artery. The decision was made to take the patient emergently for exploratory laparotomy. Once in the abdomen, a large intraperitoneal hematoma was evacuated, and after mobilization of the spleen medially, a ruptured splenic artery pseudoaneurysm was identified and controlled with multiple hemostatic stitches. The tissues surrounding the pseudoaneurysms had areas of thickened and indurated consistency, likely secondary to fibrotic changes, and had other areas with friable edematous tissue, consistent with acute on chronic versus subacute inflammation in the area. No purulent fluid was noticed. The pancreas appeared normal. Next, we proceeded with completion splenectomy and proximal ligation of the splenic artery. The specimens were sent for pathology evaluation. Operative time was 164 min. Estimated Blood Loss was 3.6 L. Patient received 7 units of packed Red Blood Cells (pRBC), 2 units of Fresh Frozen Plasma (FFP), 1 unit of platelets, 6 L of Crystalloids, and 1 L Albumin 5% intra-operatively. He did require pressure support briefly. His post-operative course was uneventful, patient recovered well, and he was discharged on post-operative day five. Final pathology demonstrated a 3.0 × 1.3 × 0.3 cm pseudoaneurysm wall and a 14 × 9.5 × 5.5 cm spleen containing multiple necrotizing granulomata, positive for Histoplasmosis species. Additional work-up performed during his hospital stay included a negative HIV test and a normal echocardiogram to evaluate for other risk factors or secondary sources of infection, respectively. Patient received post-splenectomy vaccines and was referred to Infectious Disease for follow-up, where he was prescribed Itraconazole 200 mg twice/day for 12 months. Patient was last seen at 2-month follow-up and was doing well.

## Discussion

Pseudoaneurysms (PSAs) are considered “false” aneurysms as it is consisted of only the intima and media [3, 4, 10, 15]. Pseudoaneurysms form because of injury to the vessel wall allowing blood to flow extraluminally which is contained by surrounding tissue [15]. Injury resulting in splenic artery pseudoaneurysm (SAP) formation are most commonly associated with acute and chronic pancreatitis, as well as abdominal trauma, gastric ulceration, pancreatic and gastric cancers, and infection [1–5, 8, 16–18]. When secondary to infection, they are termed mycotic pseudoaneurysms.

**Fig. 2:**
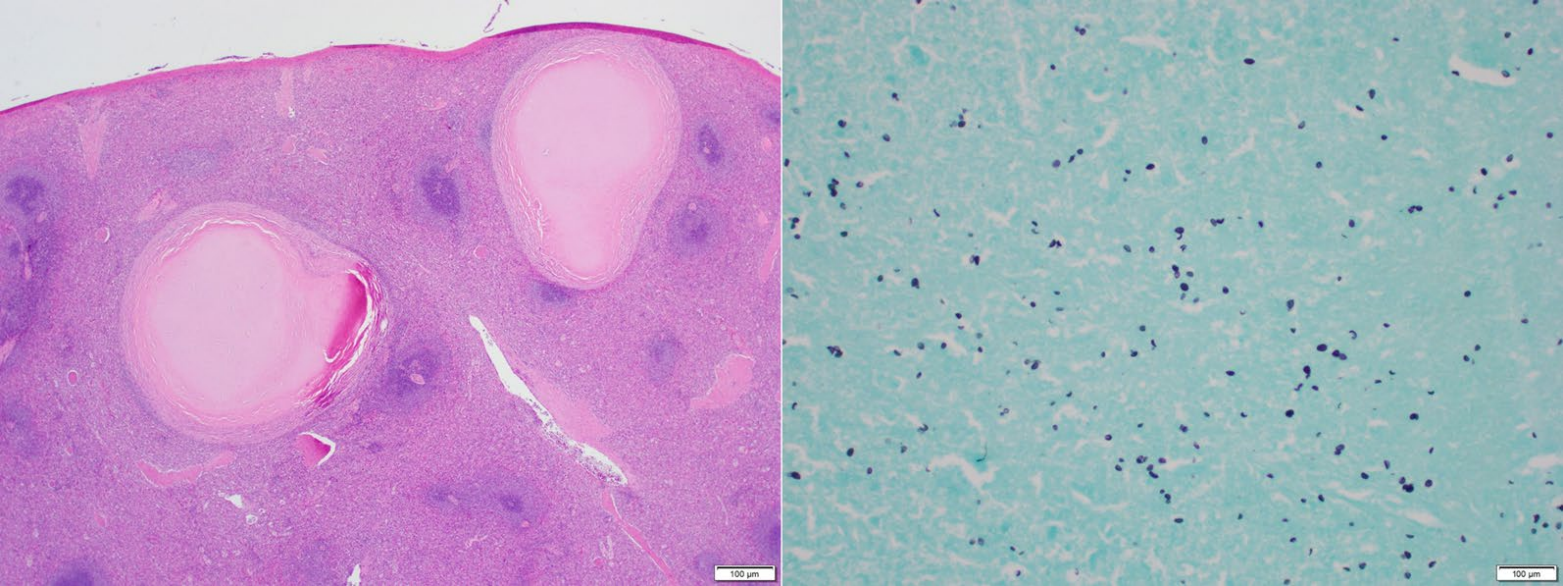
(Left) H&E stained spleen section showing multiple necrotizing granulomata (20 × magnification). (Right) GMS stain showing small, ovoid, narrow-based positive staining yeast consistent with *Histoplasmosis species*. (400 × magnification)

Mycotic pseudoaneurysmsaccount for ~ 1% of all aneurysms [15]. They develop most commonly from either intracardiac sources that cause local deposition of septic emboli, direct extension of adjacent extravascular infection or because of transient bacteremia [15]. The vascular injury that occurs is due to local destruction of the arterial wall by bacterial enzymes or serine proteases, which are a consequence of neutrophil infiltration [15]. The most common causes of mycotic pseudoaneurysms are bacterial with Staphylococcus aureus, Salmonella spp, and Pseudomonas aeruginosa, with the last being the most common [21]. However, in our case Histoplasma capsulatum was the microbe found in association with the pseudoaneurysm. Histoplasma capsulatum is a dimorphic fungus that is found largely in North America, more specifically, the Mississippi and Ohio River Valleys [22]. In immunocompetent patients, histoplasmosis is mostly a subclinical or self-limited disease that does not require treatment, however, in immunocompromised patients it can lead to disseminated or chronic pulmonary disease [22]. Fungal endarteritis is a rare disease process that can be a manifestation of disseminated histoplasmosis [23]. It has been found in cases to involve the thoracic and abdominal aorta as well as major peripheral arteries [23]. These infections are typically associated with chronic immunosuppression, diabetes, and the use of contaminated needles by drug abusers [23]. The mechanisms that fungal aneurysms evolve are presumably similar to those described for aneurysms infected by bacteria [23]. As for the patient in our case, we believe the most likely cause for seeding of Histoplasma capsulatum into the pseudoaneurysm was IV drug abuse. In addition, his echocardiogram was unremarkable and blood cultures were negative. There are a few cases reported in the literature of Histoplasma capsulatum causing or seeding aortic aneurysms [23] but to our knowledge, this is the first case describing Histoplasma capsulatum seeding a SAP (Fig. 2).

Although the splenic artery is most common visceral artery affected by pseudoaneurysms [3, 4, 19], SAPs are rare [2, 4, 8, 13]. There are less than 200 reported cases in the literature and a study out of a quaternary referral academic health care system found only 10 cases after reviewing 18 years of records [24, 25]. When they do arise, they have been shown to present in many ways: abdominal pain, nausea, hematemesis, melena, hematochezia and most concerningly, hemorrhagic shock secondary to rupture [3–6, 8, 12, 17, 25, 26]. Small splenic artery pseudoaneurysms can be asymptomatic (2.5% of cases [19]) and are usually identified as an incidental finding on imaging [3, 7, 8]. However, larger pseudoaneurysms (> 5 cm), termed “giant pseudoaneurysms”, are commonly symptomatic, although the risk of rupture seems not to be related to the pseudoaneurysm size [7, 10, 20, 25, 27]. SAPs may be detected on exam as a pulsatile mass in the upper left quadrant or epigastric region with associated left upper quadrant pain [3, 4, 28]. If ruptured, they can bleed into the stomach, duodenum, lesser sac, pancreatic duct (hemosuccus pancreaticus), colon, or directly into the peritoneal cavity leading to the symptoms described above [9, 24, 25, 28, 29]. In our case, the patient presented with acute onset diffuse abdominal pain associated with peritonitis, hypovolemia, and signs of impending hemorrhagic shock.

It is important to treat all SAPs appropriately due to its risk of rupture resulting in hemorrhagic shock and death [3, 4, 8, 9, 13]. Ultrasound, contrast enhanced ultrasound (CEUS), computed tomography (CT) and magnetic resonance imaging (MRI) are imaging modalities that can play a role in diagnosing a SAP, however CT angiography (CTA) is the gold standard for diagnosis [3, 4, 8–10, 27]. As described above, our patient underwent a CT scan of the abdomen and pelvis with intravenous contrast which demonstrated active extravasation of contrast from the splenic artery into the splenic hilum, consistent with a ruptured SAP.

The risk of rupture of an SAP has been estimated to be near 37%, with a mortality rate of 90% if left untreated [5, 10, 14, 20]. The hemodynamic status of these patients, location of the SAP, and risk of organ ischemia helps to guide its management.. [2, 3, 13, 28, 30]. Historically, open or laparoscopic surgical arterial ligation and splenectomy with or without distal pancreatectomy was the treatment of choice with an excellent success rate, however, it is associated with an increased risk of morbidity and mortality (9% and 1.3%, respectively) [3, 20]. More recently, transcatheter arterial embolization (TAE) has become the standard treatment option for pseudoaneurysms [7, 20, 26] in hemodynamically stable patients [4, 9, 10, 12, 20], due to its lower morbidity and mortality and high success rate [3, 4, 7, 10, 13]. In unstable patients [2], those who fail embolization [16], or those with persistent or re-bleeding after a TAE procedure, surgery is indicated [4, 9, 12, 24]. As described above, our patient did not undergo TAE for his diagnosed SAP rupture due to patient being relatively unstable and the interventional radiologist’s assessment of low probability of success given the location, extension, and severity of the active bleeding. Therefore, this patient was taken to the operating room for an emergent exploratory laparotomy where hematoma evacuation, splenectomy, and proximal ligation of the splenic artery were performed.

Finally, it is important to provide adequate follow-up and treatment of fungal infections related to mycotic pseudoaneurysms to prevent further dissemination, disease progression, or recurrence of pseudoaneurysms. Similar consideration should be given to associated comorbidities and risk factors (i.e.. management of immunosuppressive disorders, addressing IV drug abuse). Post-operatively, our patient was started on Itraconazole 200 mg twice/day for 12 months and will continue to follow-up with infectious disease specialists.

## Conclusion

Splenic artery pseudoaneurysms are rare but require prompt workup and treatment. SAPs should be on the differential diagnoses when a patient presents with abdominal pain, hemoperitoneum, hematemesis, or hematochezia in the setting of pancreatitis, peptic ulcer disease, gastric and pancreatic cancers, and IV drug abuse. Patients with fungal causes of mycotic pseudoaneurysms are likely immunocompromised and should be managed appropriately in the peri and postoperative setting. To our knowledge, this is the first reported case of ruptured mycotic SAP secondary to Histoplasma capsulatum infection.

## Data Availability

Not applicable.

## Abbreviations

PSA: Pseudoaneurysm
SAP: Splenic artery pseudoaneurysm
CTA: Computed tomography angiography
MRI: Magnetic resonance imaging
TAE: Transcatheter arterial embolization
